# Supplementary material for: Co-occurrences enhance our understanding of aquatic fungal metacommunity assembly and reveal potential host–parasite interactions
Source: FEMS Microbiol Ecol. 2022 Oct 6;98(11):fiac120. doi: 10.1093/femsec/fiac120 (PMC9621394; doi:10.1093/femsec/fiac120)
Supplement: fiac120_Supplemental_Files [file fiac120_supplemental_files.zip › Supp_data.pdf]

<u>Supplementary Material for:</u> Co-occurrences enhance our understanding of aquatic fungal metacommunity assembly and reveal potential host–parasite interactions

Máté Vass[1]*, Karolina Eriksson[1], Ulla Carlsson-Graner[1], Johan Wikner[1,2], Agneta Andersson[1,2]

[1]Department of Ecology and Environmental Science, Umeå University, Umeå, Sweden
[2]Umeå Marine Sciences Centre, Umeå University, Hörnefors, Sweden
*corresponding author's address: mate.vass@umu.se, Umeå University, Linnaeus väg 6, 901 87, Umeå, Sweden

Amplification details:
PCR was performed in 40 µL reactions with 1.5 µL PrimeStar GXL polymerase (#R050A; Takara), 12 pmol of barcoded primer (see list of barcodes in Supplementary Table S1), 1 mM dNTPs, 8 µL 5× PrimeSTAR GXL Buffer and 2 µL of DNA template (0.9–49.6 ng/µL). Amplifications were done on a Bio-Rad T100 Thermal Cycler (Bio-Rad Laboratories) with an initial denaturation at 98 °C for 1 min, then 36 cycles at 98 °C for 10 s, annealing at 55 (or 60) °C for 15 sec and elongation at 68 °C for 4 min.

Sequencing details:
Amplicon library was end-repaired and adapted for nanopore sequencing using NEBNext Companion Module for Oxford Nanopore Technologies Ligation Sequencing (#E7180S), finally, a clean-up step was performed to enrich for amplicon length of > 3 kb using the Long Fragment Buffer (LFB) provided within the Ligation Kit. Thanks to the real-time monitoring during sequencing, we noticed a low pore occupancy (e.g., high Strand:Single Pore ratio), hence, we prepared the remaining library with extended incubation times (+5 and +10 mins during end-prep. and adapter ligation, respectively) and loaded it to the existing run (55.14 fmol DNA in total).

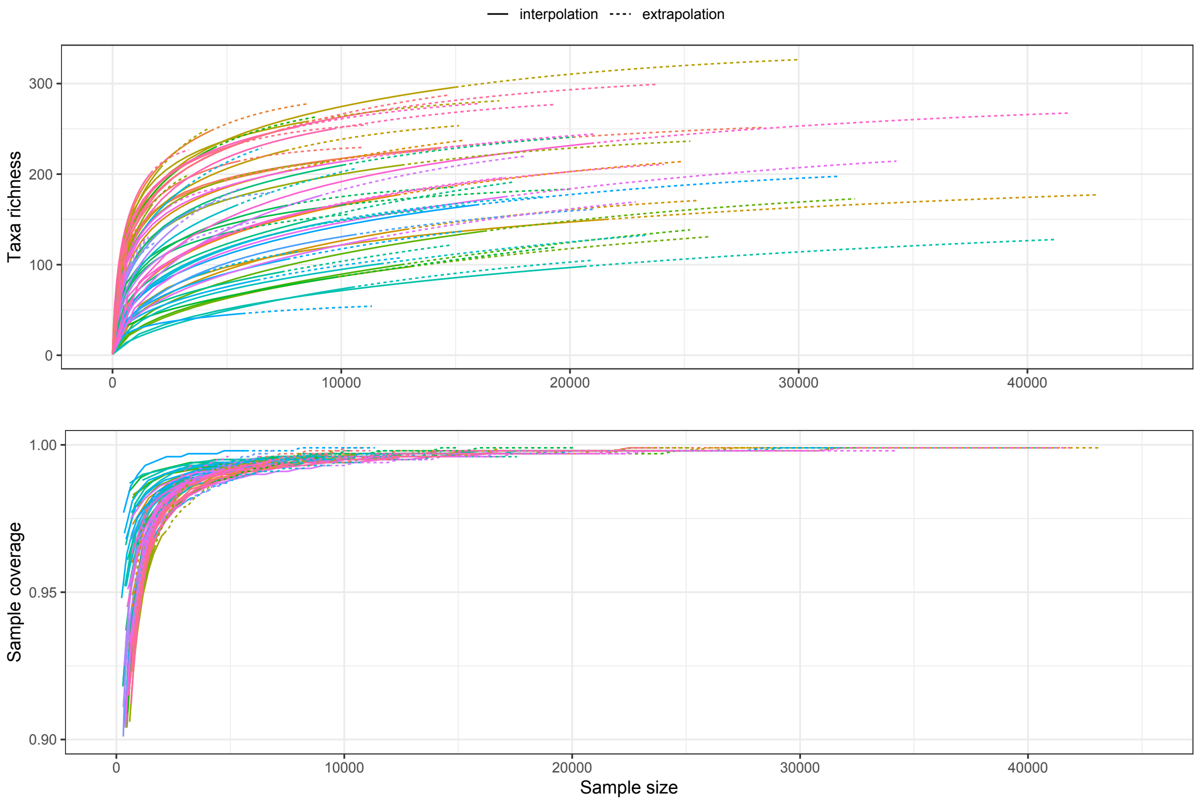

Figure S1. Diversity estimates and sample completeness (coverage) curves.

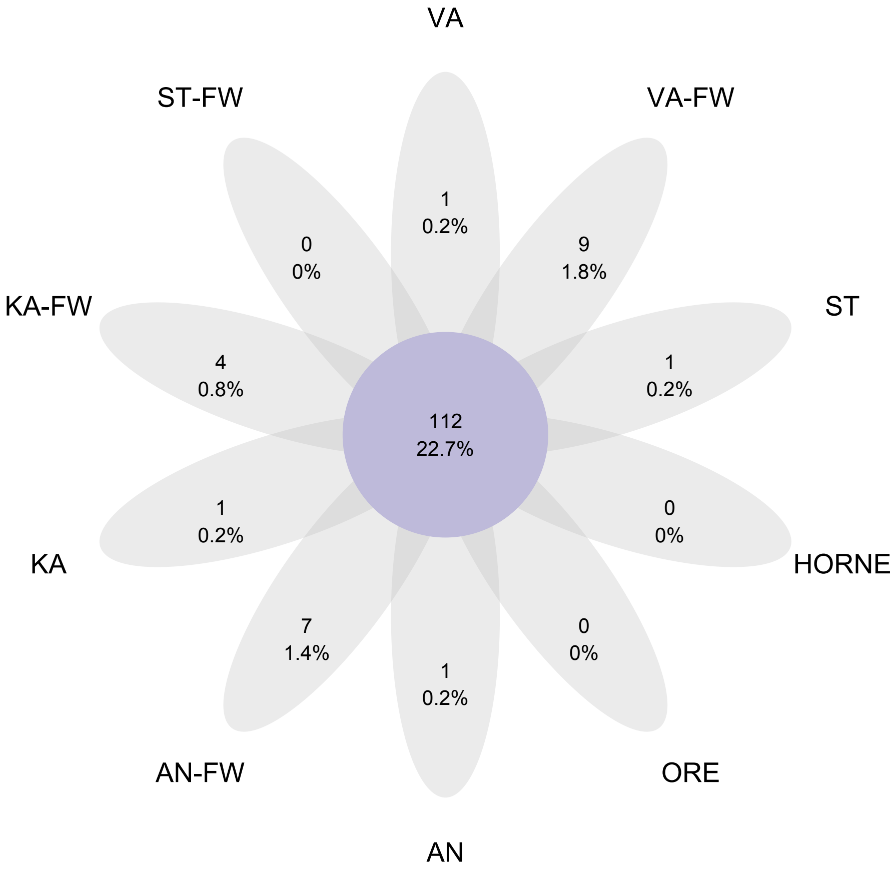

Figure S2. Venn diagram representing the unique and shared fungal taxa in the sampled bays and their freshwater inlets ('-FW' tag), or in the offshore sites (HORNE, ORE). For code descriptions, see Figure 1. in the main text.

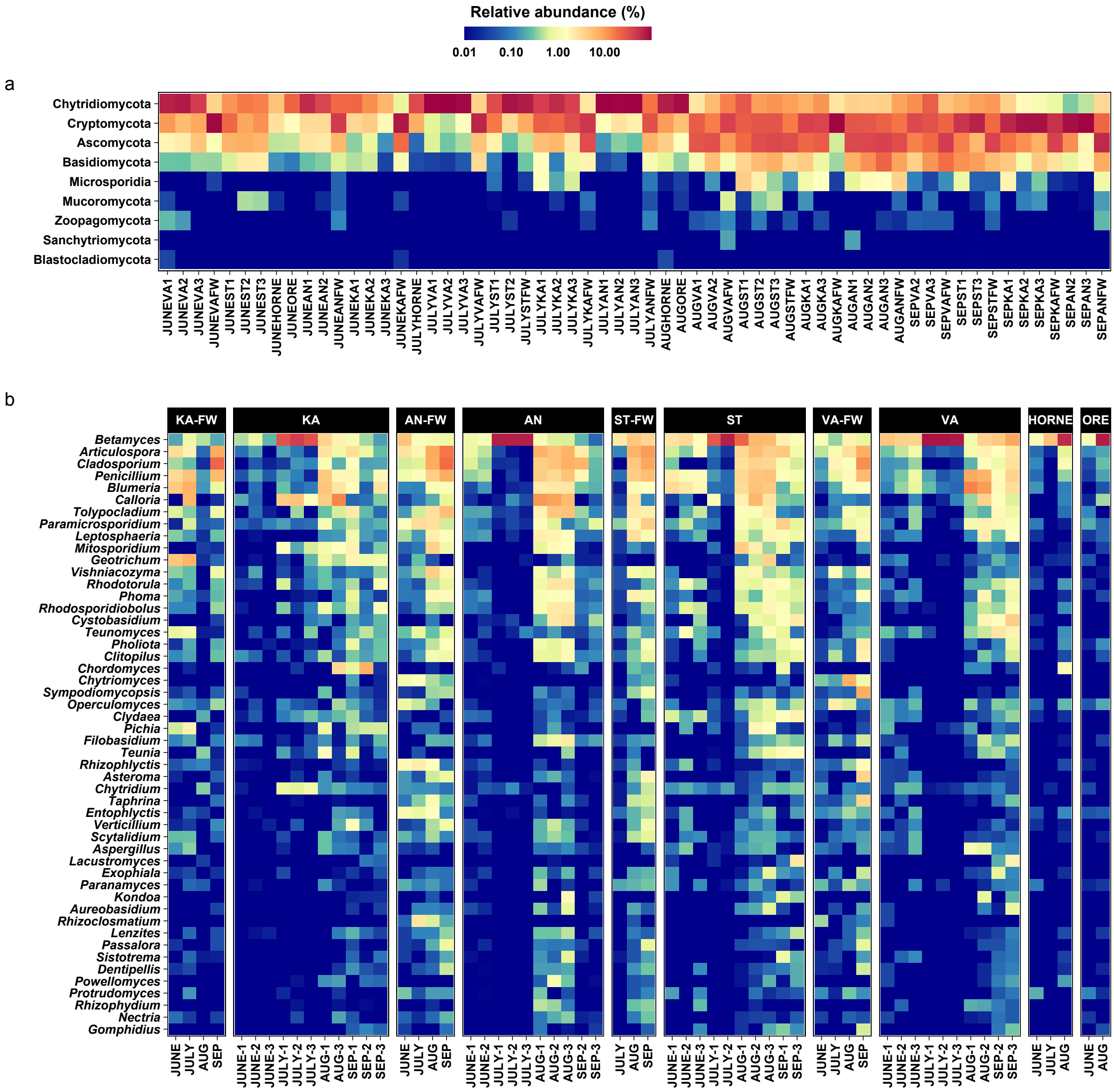

Figure S3: Heatmaps showing the temporal changes of fungal communities at (a) phylum and (b) genus level. Note that only the top 50 OTUs assigned down to genus level (BLAST-based taxonomy against the NCBI *nt* database) are listed.

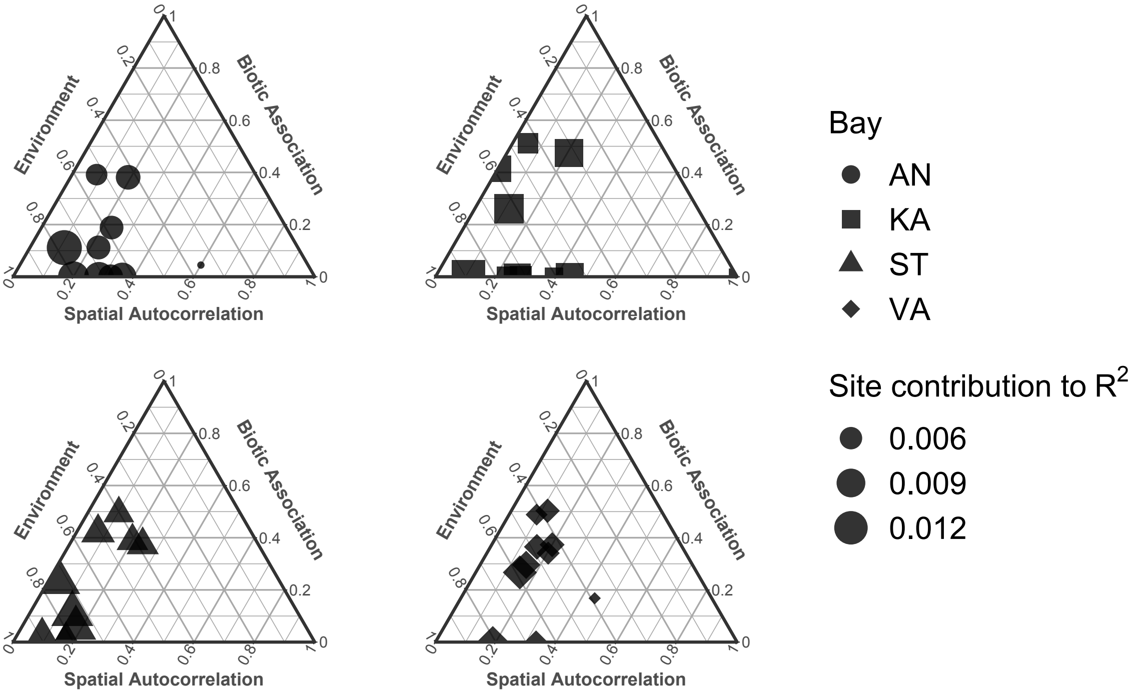

Figure S4. Site-specific internal metacommunity structure. The ternary plots describe the contributions of space, environment and biotic associations to the metacommunity level properties among the four bays. The size of the symbols refers to the variation explained by the model (R^2) for each site.

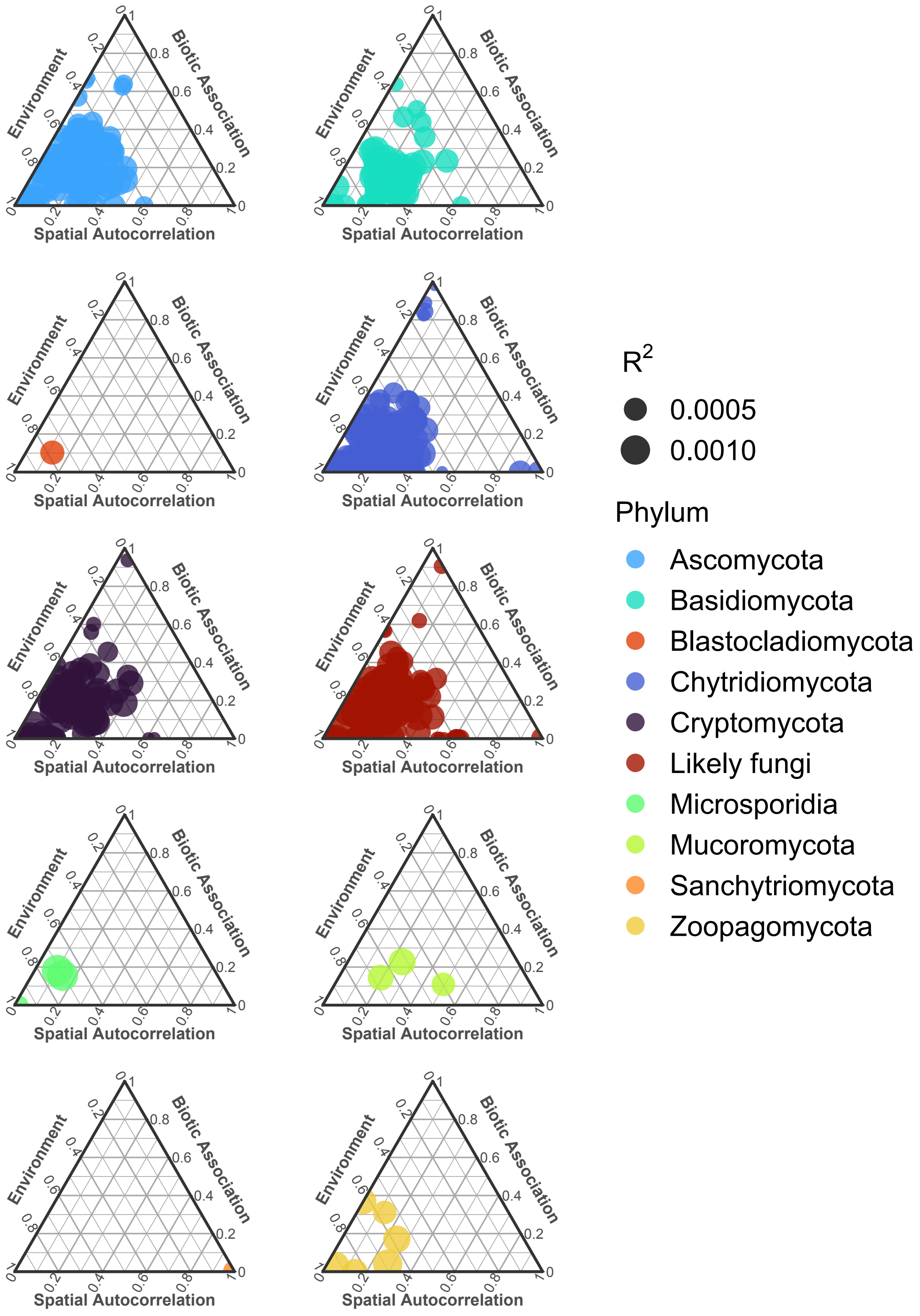

Figure S5. Taxa-specific internal metacommunity structure. The ternary plots display the relative influence of environmental conditions, space and biotic associations on the distribution of each fungal taxon (OTU). The size of the symbols refers to the variation explained (R^2) by the model for each OTU.

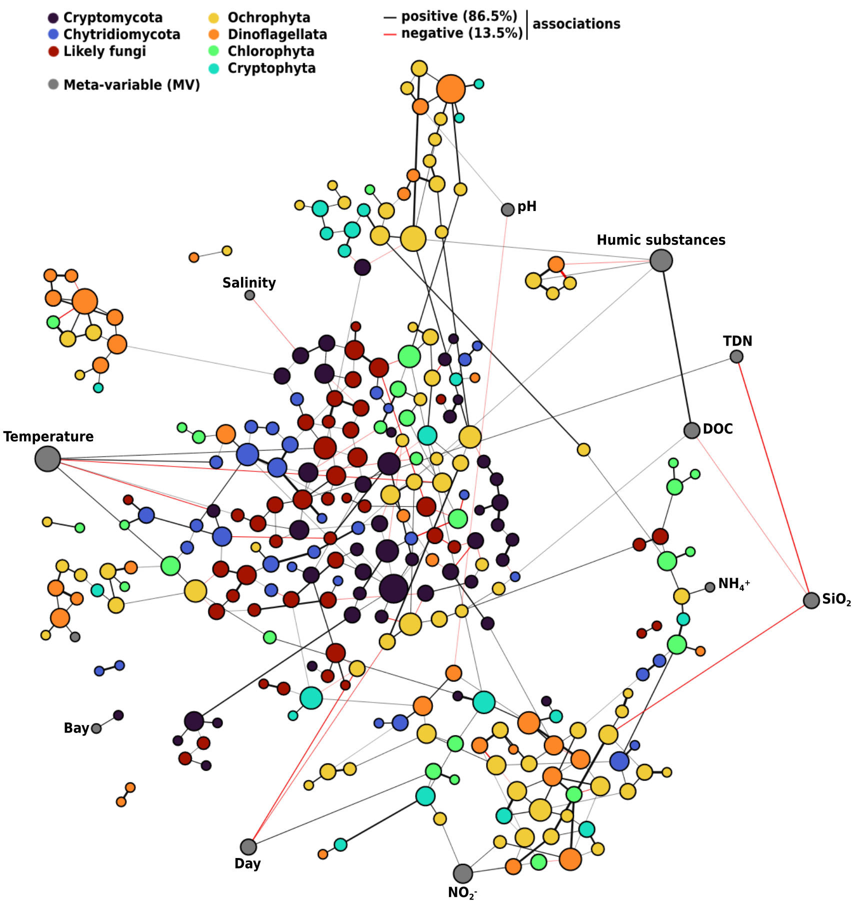

Figure S6. Prediction of fungi–algae interactions with FlashWeave ($p < 0.01$). The size of nodes (OTUs or meta-variables; n = 288) are proportional to the number of predicted interactions (degree). Edge width refers to the strength of correlations (weight cutoff > |0.4|; n = 364) and coloured by association type (positive or negative). 'Likely fungi' refers to fungal OTUs without matches to any phylum (BLAST-based taxonomy against NCBI *nt* database).
